# Biology and Role of Extracellular Vesicles (EVs) in the Pathogenesis of Thrombosis

**DOI:** 10.3390/ijms20112840

**Published:** 2019-06-11

**Authors:** Marta Zarà, Gianni Francesco Guidetti, Marina Camera, Ilaria Canobbio, Patrizia Amadio, Mauro Torti, Elena Tremoli, Silvia Stella Barbieri

**Affiliations:** 1 Unit of Heart-Brain Axis: Cellular and Molecular Mechanisms, Centro Cardiologico Monzino IRCCS, 20138 Milano, Italy; marta.zara@ccfm.it (M.Z.); patrizia.amadio@ccfm.it (P.A.); 2 Department of Biology and Biotechnology, University of Pavia, 27100 Pavia, Italy; gianni.guidetti@unipv.it (G.F.G.); ilaria.canobbio@unipv.it (I.C.); mtorti@unipv.it (M.T.); 3 Department of Pharmacological and Biomolecular Sciences, University of Milan, 20133 Milano, Italy; marina.camera@unimi.it; 4 Unit of Cell and Molecular Biology in Cardiovascular Diseases, Centro Cardiologico Monzino IRCCS, 20138 Milano, Italy; 5 Scientific Direction, Centro Cardiologico Monzino IRCCS, 20138 Milano, Italy; elena.tremoli@ccfm.it

**Keywords:** Extracellular vesicles, microvesicles, exosomes, biomarker, arterial thrombosis, venous thrombosis

## Abstract

Extracellular vesicles (EVs) are well-established mediators of cell-to-cell communication. EVs can be released by every cell type and they can be classified into three major groups according to their biogenesis, dimension, density, and predominant protein markers: exosomes, microvesicles, and apoptotic bodies. During their formation, EVs associate with specific cargo from their parental cell that can include RNAs, free fatty acids, surface receptors, and proteins. The biological function of EVs is to maintain cellular and tissue homeostasis by transferring critical biological cargos to distal or neighboring recipient cells. On the other hand, their role in intercellular communication may also contribute to the pathogenesis of several diseases, including thrombosis. More recently, their physiological and biochemical properties have suggested their use as a therapeutic tool in tissue regeneration as well as a novel option for drug delivery. In this review, we will summarize the impact of EVs released from blood and vascular cells in arterial and venous thrombosis, describing the mechanisms by which EVs affect thrombosis and their potential clinical applications.

## 1. Introduction

Arterial and venous thrombosis are the leading causes of mortality and morbidity worldwide and the underlying mechanisms at the basis of these thrombotic conditions are not completely known [1]. Arterial and venous thrombosis are classically considered as distinct diseases, differing for risk factors, pathophysiology, and clinical manifestations. Arterial thrombosis mainly encompasses myocardial infarction (MI) and ischemic stroke; it usually originates as a consequence of advanced atherosclerotic lesions that, upon plaque rupture, leads to clot formation and vessel occlusion. Arterial thrombi are mainly composed of platelets and are usually defined as white thrombi. Classical risk factors for atherosclerosis and subsequent arterial thrombosis are smoking, hypertension, diabetes, obesity, and hyperlipidemia. By contrast, venous thromboembolism (VTE) occurs as the result of disturbed blood flow or stasis, hypercoagulation, or endothelial dysfunction due to vessel injury or inflammation. The leading forms of VTE are deep vein thrombosis (DVT), mostly involving legs, and pulmonary embolism (PE). Venous thrombosis leads to the formation of thrombi rich in fibrin and erythrocytes, giving them the appearance of red thrombi [2]. Every condition causing hypercoagulation (e.g., cancer), endothelial activation (like surgery or trauma), or blood stasis are considered risk factors for developing VTE [3,4].

**Figure 1:**
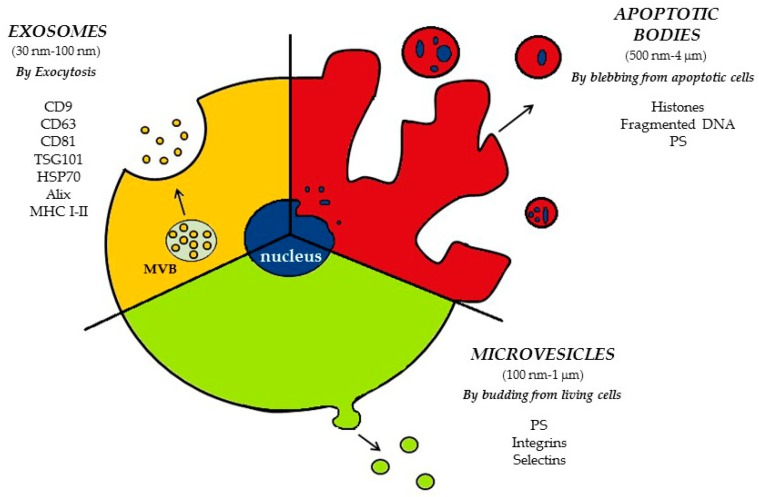
Mechanisms of extracellular vesicle (EV) release and their specific markers. Apoptotic bodies (ApoBDs) are released by membrane blebbing of apoptotic cells; microvesicles (MVs) bud directly from the plasma membrane of living cells, while exosomes are formed as the intraluminal vesicles (ILVs) by budding into early endosomes and multivesicular bodies (MVBs) and are released by exocytosis. PS: phosphatidylserine; MHC: major histocompatibility complex; TSG101: tumor susceptibility gene 101 protein; HSP70: heat shock 70 kDa protein.

Extracellular vesicles (EVs) are small membrane vesicles released from different types of activated or apoptotic cells, including leukocytes, platelets, erythrocytes, and endothelial cells, detected both in human and animal body fluids. EVs have been classified into three major populations (apoptotic bodies, microvesicles, and exosomes) characterized by size and origin (Figure 1). Among the different types of EVs, an overlapping in biophysical characteristics, including the size range and protein expression, have been reported. However, selective markers of exosomes, microvesicles, and apoptotic bodies have been identified [5].

EVs, acting as a reservoir of lipids, proteins, nucleic acids including microRNAs and noncoding RNA, play important roles in physiological and pathological conditions contributing to coagulation and inflammation, as well as to intercellular communication [5].

Over the past few years, particular attention has been paid to EVs as modulators of inflammation, vascular dysfunction, and thrombosis [6,7]. Since EVs retain surface and cytosolic proteins expressed in their parental cells, they represent an attractive diagnostic tool for a noninvasive liquid biopsy. However, their use as biomarkers in real clinical practice is actually hampered by technical challenges in their isolation, quantification, and characterization [7,8,9]. Recently, to overcome these problems, different manuscripts, including position papers by International Society on Thrombosis and Haemostasis (ISTH), European Society of Cardiology (ESC), and International Society for Extracellular Vesicles (ISEV), have provided a set of recommendations for the isolation and characterization of EVs [7,8,9,10,11].

In this review, we summarize the impact of EVs released from blood and vascular cells in arterial and venous thrombosis, describing the underlying mechanisms and their potential clinical applications.

## 2. Classification and Biogenesis of EVs

EVs are membrane-surrounded particles released by most eukaryotic cell types, whose size varies from about 30 nm up to a few µm. In humans, EVs can be found in many bodily fluids, including blood, urine, tears, and saliva, and are involved in several physiological and pathological processes, including cardiovascular diseases [6].

Because of their heterogeneity, the classification of EVs is complex and the study of the different populations and subpopulations of EVs is challenging. In particular, the purification and the detection of EVs represent a very important issue.

Depending on the biological source and on the class of EVs, different protocols for their purification and analysis have been proposed. Differential centrifugation, ultracentrifugation, density gradient centrifugation, precipitation, ultrafiltration, size exclusion chromatography, and affinity chromatography can be adopted for the isolation of EVs [8]. However, although excellent reviews and methodological guidelines are available on this topic [8,9,10,11,12,13,14,15] (Table 1), the purification of EVs still represents a critical aspect. This is mainly due to the small size of vesicles and to the difficulty in depleting sample from non vesicle contaminants, such as protein aggregates, lipoproteins, and cell organelles. In addition, the preanalytical steps may influence the outcome of the analyses and a careful methodological standardization is still required to obtain reliable information.

As for their isolation, EVs characterization represents a challenging task. The morphology of different types of EVs was characterized mostly by transmission electron microscopy (TEM) and, more recently, by Cryo-TEM. The shape of EVs from different biological sources is heterogeneous and up to nine different classes have been identified, such as single spherical vesicles, double-membrane vesicles, oval vesicles, vesicles within vesicles, tubules, and others [12,16,17,18]. Morphological classification of EVs is not widely adopted, particularly because vesicle isolation and electron microscopy analyses are prone to artifacts.

Atomic force microscopy (AFM) gives information concerning the very broad size distribution of the EVs, calculating their diameter. AFM has the advantage of minimizing sample preparation by measuring EVs in their native conditions [19].

Size and concentration of vesicles may also be examined by dynamic light scattering (DLS) and by nanoparticle tracking analysis (NTA) that take advantage of the ability of EVs to move under Brownian motion in the liquid phase [19]. NTA provides quantitative and qualitative information of EVs when it is used in fluorescent mode [20]. In addition, the characterization of large vesicles has been mostly performed by flow cytometry. This methodology is based on the analysis of EVs suspended in a fluid and it allows to quantify and classify the vesicles according to antigen expression levels by using fluorochrome-conjugated antibodies [19].

Nowadays, a combination of the methods mentioned above and multiomic analyses is the preferential approach for EVs characterization (see Table 1 for a summary).

Through these methods, information about the size, the shape, and the cargo of EVs can be collected, allowing the determination of their cellular and subcellular origin. Accordingly, the current classification is based on EVs biogenesis and three different classes of EVs are now normally distinguished: apoptotic bodies, microvesicles, and exosomes, which originate through distinct mechanisms and are involved in different cellular processes [5].

### 2.1. Apoptotic Bodies (ApoBDs)

Apoptotic bodies (ApoBDs) are organelle-containing EVs ranging from 500 nm to 4 µm in diameter and represent the main type of EVs released during programmed cell death. In the late phases of apoptosis, the content of the dying cell is packed into ApoBDs, which are then released by membrane blebbing and eventually engulfed by phagocytic cells [21]. Such cell fragmentation into ApoBDs is involved in the clearance of the dying cell [22], but ApoBDs also promote intercellular communication by delivering their content into recipient cells [23,24]. The formation of ApoBDs by dying cells influences immune regulation and plays important roles in different pathological conditions, including autoimmunity and cancer [25].

### 2.2. Microvesicles (MVs)

Microvesicles (MVs) are from 100 nm to 1 µm in size, are shed by the plasma membrane of living cells, and are also known as microparticles, oncosomes, and ectosomes, depending on the cellular source or the field of investigation [17]. MVs were first identified in 1967 by Peter Wolf, who described procoagulant derivatives of platelets, originally named as “platelet dust” [26], now typically referred to as platelet-derived microparticles (PMPs).

MVs are released by budding of the plasma membrane through a mechanism supported by cytoskeletal remodeling. Several molecules are selectively loaded in the growing vesicle including lipids, such as cholesterol and ceramide, nucleic acids, including mRNA and miRNA, proteins, and other bioactive molecules. MVs also express specific plasma membrane surface antigens, which allow the precise identification of their cellular origin.

The mechanism of MV biogenesis requires complex machinery to support the trafficking of cargo, segregation of lipids and vesicle fission. Changes of local lipid composition and the recruitment of phospholipid translocases contribute to the curvature of the plasma membrane, thus facilitating the generation of the vesicles [27]. Recent studies have demonstrated that components of the endosomal sorting complex required for transport (ESCRT), which play major roles in endosomal trafficking and biogenesis of exosomes, also participate to the release of MVs [28]. Tumor susceptibility gene 101 (TSG101), a component of the ESCRT complex I, supports vesicle budding through the interaction with arrestin domain-containing protein-1 (ARRDC1). Moreover, ESCRT complex III is involved in the late phases of MV biogenesis and mediates the detachment of the vesicle from the plasma membrane [29].

Cargo proteins are selected through different mechanisms. Plasma membrane anchors, such as myristoylation and palmitoylation, contribute to targeting highly oligomeric protein to the domains of MVs budding. Moreover, different proteins, including Adenosine diphosphate-ribosylation factor 6 (ARF6), Ras-related protein 22a, vesicle-soluble NSF attachment protein receptor, Vesicle-associated membrane protein 3, T-cell internal antigen 1, and Argonaute2, have been involved in the trafficking of proteins and nucleic acids to MVs, depending on the cellular context and the stimulus that induced vesiculation [30].

The fission of MVs and their release from the plasma membrane is supported by ATP-dependent contraction of actin/myosin cytoskeletal structures sustained by an increase in the cytosolic concentration of Ca^2+^ [28,30]. In addition to trafficking control, ARF6 also regulates this contractile machinery responsible for MV fission. ARF6 controls the localization of myosin light chain kinase (MLCK), via the activation of ERK and phospholipase D (PLD), leading to the phosphorylation of myosin light chain (MLC) and the activation of cytoskeletal contraction in the region of vesicle budding [31].

### 2.3. Exosomes

Exosomes and MVs, although being characterized by similar global architecture, present several major differences, particularly regarding the mechanism of formation. Exosomes are released by cells on the exocytosis of a specific type of late endosome named multivesicular body (MVB). MVBs contain preformed exosomes, defined as intraluminal vesicles (ILVs) before exocytosis, that are generated by intraluminal budding of the membrane of late endosomes [28]. Exosomes are smaller than MVs (about 30 to 100 nm in size), and because of their endosomal origin, they typically contain proteins involved in membrane transport and fusion, components of ESCRT, and tetraspanins (CD63, CD9, and CD81) [32].

The formation of ILVs during the conversion of late endosomes to MVBs involves the accumulation at the level of the forming vesicle of different lipids, including cholesterol, sphingomyelins, and particularly ceramide, which plays important roles in vesicle budding [33,34]. The growing ILV is loaded with proteins, lipids, and RNA. Ubiquitination has been proposed as a key mechanism to target specific proteins to ILVs and ubiquitinated proteins are delivered to the vesicle by the combined action of the four ESCRT complexes and the associated AAA ATPase Vps4 complex [28,35,36]. In addition to the selection of the cargo, the ESCRT complexes also regulate the membrane-remodeling and the scission required for ILV budding [28]. Nonetheless, the functionality of ESCRT is not mandatory for vesicle formation and subsets of ILVs can be generated in an ESCRT-independent, lipid-driven fashion [35,36,37]. The vesicle pinching off in the lumen of MVB is sustained by the polymerization of the actin cytoskeleton, which in turn is regulated by several small GTPases, including ARF6, Cdc42, and Rab-family members. The last step of exosomes biogenesis, the exocytosis of ILVs, is still poorly understood, but a few molecular players involved in this process have been identified, including the small GTPases of the Rab family (Rab11, Rab27, and Rab35) and the SNARE complex [28].

## 3. EVs in Intercellular Communication

EVs released into extracellular space can transfer information to neighboring or distal cells and deliver their contents inducing functional response and promoting physio-pathological changes. EVs act as a delivery system for bioactive proteins, lipids, mRNA, miRNA long noncoding RNA and occasionally genomic DNA protecting their cargo from degradation in plasma. Then, EVs may transfer genetic information inducing transient or persistent modifications in recipient cells [38,39], suggesting their potential use in tissue regeneration and human gene therapy. EVs may act not only as paracrine/endocrine effectors, but autocrine responses have been also described [40].

EVs selectively adhere to recipient cells through interaction with specific lipids or ligand receptors (e.g., tetraspanins, integrins, lipids, lectines, heparin sulfate proteoglycans, and extracellular matrix) and directly stimulate them [41]. In addition, it has been shown that the binding between phosphatidylserine exposed on the surface of certain EVs and annexin V blocked the fusion of monocyte-derived EVs with activated platelets [41].

EVs can be internalized into recipient cells through several mechanisms including micropinocytosis, phagocytosis, and endocytosis via lipid rafts, clathrin and caveolae, or through the direct membrane fusion. However, it should be added that in similar experimental setting has been shown that clathrin-independent, caveolin 1-dependent endocytosis may have both negative and positive effects in EV uptake [42,43,44].

Internalized EVs are included into multivesicular endosomes (MVEs), where they are mixed with endogenous intraluminal vesicles. Then, MVEs are targeted to lysosomes for degradation of EV proteins and lipids that will represent a source of metabolites to recipient cells. Otherwise, MVEs are docked to the plasma membrane, or trough back fusion they release their contents into the cytoplasm [45].

Through all these mechanisms, EVs may affect the behavior of recipient cells. P-selectin expressed on PMPs binds leukocyte glycoprotein ligand-1 (PSGL-1), promoting leukocyte aggregation and accumulation [46].

EVs can also transfer functional receptors either to target cells that originally do not express them or they may contribute to enhance the number of expressed receptors into recipient cells. For instance, PMPs may transfer platelet antigens (CD41, CD61, CD62, CXCR4, PAR-1) to hematopoietic stem-progenitor cells (HSPCs) promoting their proliferation and adhesion [47].

Interestingly, arachidonic acid, PAF-like lipids as well as lipoxygenase products transferred to recipient cells by EVs participate to amplification or modulation of thrombus formation. In particular, these EVs promoted the adhesion of monocytes to endothelium, and stimulate prostacyclin synthesis or thromboxane A2 production if metabolized by endothelial cells or platelets, respectively [48].

EVs, by transferring specific mRNA, stimulate angiogenesis in quiescent endothelial cells when released from endothelial progenitor cells [49], and differentiation of progenitors in functional megakaryocytes when released from mature megakaryocytes [50]. Finally, transcellular transfer of EVs enriched in miRNAs conferred both a proinflammatory and anti-inflammatory phenotype to immune cells [51], suggesting their complex and critical role in physiological and pathological function.

In addition, several studies have focused on the role of miRNA-carrying EVs including exosomes in the modulation of tissue regeneration. However, the mechanism by which exosome signaling affects cell function and tissue repair is still ambiguous and not completely elucidated [52].

Recently, miRNome analyses of exosomes isolated from human platelets showed high expression of miR126-3p, mi-R21, mi-223, miR-339, miR-328, miR-22, miR-185, miR-320b. Then, the miR126-3p present in PMPs was internalized by human macrophages and, by suppressing expression of different genes, increased the phagocytic ability of these cells [53]. Similarly, platelets exosomes enriched in miR 21, miR-223, and miR-339 modulated platelet-derived growth factor receptor-beta in vascular smooth muscle cells, regulating their phenotypic modification [54]. In addition, the miR-223 promoted the apoptosis of endothelial cell through insulin-like growth factor 1 receptor affecting atherosclerotic plaque progression [52]. However, the information on the platelet-regulated exosomal trafficking is still limited.

## 4. Circulating EVs

EVs detected in the plasma of healthy subjects are derived mainly from platelets and/or megakaryocytes, and a minority originate from erythrocytes, leukocytes, and endothelial cells. Interestingly, the levels of these classes of EVs change under pathological conditions [12,55,56,57,58].

### 4.1. Megakaryocyte and Platelet-Derived Vesicles

Platelets release preferentially two types of EVs: microvesicles, commonly known as platelet-derived microparticles (PMPs), and exosomes. Recently, it has been demonstrated that platelets undergo apoptosis [59] thus suggesting that they may also release apoptotic bodies.

**Figure 2:**
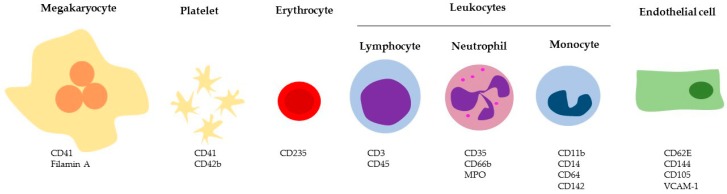
Specific cellular markers of EVs. MPO: myeloperoxidase; VCAM-1 Vascular cell adhesion protein 1.

PMPs express specific platelet markers such as CD41 and CD42b [56] (Figure 2).

The generated PMP population is heterogeneous in terms of size, structure, and protein expression, depending on the cell-activating stimulus [60,61].

PMPs are released from platelets upon activation with different physiological agonists such as lipopolysaccharide (LPS) from bacteria, viruses, cancer cells, upon shear stress, during platelet storage, and cryopreservation [62,63,64,65,66]. PMPs are important in cell–cell communication, as they transport and deliver bioactive molecules, receptors, functional enzymes, cytosolic proteins, cytokines, microRNA (miRNA), and noncoding RNA throughout the body [67].

A growing body of evidence demonstrates that megakaryocytes also release MVs. The first evidence dates back to 1997 when the group of Cramer observed that cultured human megakaryocytes shed MVs expressing integrin αIIbβ3 (CD41) on their surface [68]. This first observation was later confirmed by high-resolution microscopy revealing the release of MVs from cultured murine and human megakaryocytes [69,70].

MVs released from megakaryocytes share with PMPs the expression of typical markers such as CD41, CD42b, and GPVI [69] but not markers of platelet activation, such as P-selectin (CD62P). In contrast, full-length filamin A was found in megakaryocytes-derived MVs, but not in CD62P positive PMPs [69,71] (Figure 2 and Table 2).

Both megakaryocytes and platelets release MVs that express tissue factor (TF) and phosphatidylserine (PS) [72,73,74]. PMPs are highly prothrombotic, support thrombin generation and thrombus formation [75], and are elevated in several diseases associated with platelet dysfunctions [76,77,78,79].

Interestingly, using electron microscopy and annexin V-conjugated gold nanobeads, two populations of PMPs were identified: about half of PMPs detected expose anionic PS on their surface and thus are positive for annexin V [12], and the remaining are negative for PS. The subset of annexin V-positive PMPs has procoagulant activity and is rapidly removed from the circulation, whereas the annexin V-negative PMPs have distinct roles other than thrombosis, circulate for a longer period and participate to intercellular communication [80].

Platelet exosomes derive from MVBs and α-granules [81]. In addition to typical exosome markers, platelet exosomes are enriched in CD41 [82] and in specific miRNA, including miR-21, miR-223, and miR-339 [83,84] (Table 2). These miRNAs may influence the behavior of targeted cells and have both been linked to numerous human diseases [85]. Proteomic analysis of platelet exosomes revealed the presence of HSP70, GPIb, GPV, and WNT glycoproteins which regulate WNT signaling in monocytes and endothelial cells [86]. Interestingly, exosomes released from platelets stimulated with thrombin and collagen present an increased amount of chemokines CXCL4, CXCL7, and cytoplasmic high-mobility group box 1 (HMGB1) protein, which contribute to the pathogenesis of atherosclerosis [87] (Table 2).

### 4.2. Erythrocyte-Derived Vesicles

Erythrocytes or red blood cells are the most abundant cells and are able to release both microvesicles and exosomes [88].

Erythrocytes-derived MVs and exosomes are positive for the typical markers of this cell population (e.g., CD235a) (Figure 2) [89] and are released upon the increase of intracellular calcium concentration, PKC activation [90], and osmotic shock. MV formation is an integral part of erythrocyte homeostasis and it is responsible for the loss of 20% of the cell membrane and hemoglobin during physiological aging [91]. In vivo erythrocyte-MVs contain membrane proteins band 3, actin, hemoglobin A, the EVs markers ALIX and TSG101, and complement-inhibiting proteins CD55 and CD59 (Table 2). These EVs are enriched in enzymes involved in redox homeostasis and have large amounts of complement proteins and immunoglobulins, and, like erythrocytes, carry a great amount of iron [92] (Table 2). Moreover, most erythrocyte-MVs express PS on their surface and therefore are procoagulant [93].

MVs from erythrocytes are free of nuclear and mitochondrial DNA, therefore they have been proposed as a useful tool to deliver RNA-based therapies [94].

Little information is available about the generation and function of erythrocyte-derived exosomes, but it has been suggested that they are formed during reticulocytes maturation or in stored erythrocyte units [95,96]. Erythrocyte-derived exosomes, in addition to CD235a, express also CD63, they are capable to stimulate monocytes to produce TNF-α and to augment T-cell proliferation, which suggests a potential role in the inflammatory and immune response.

### 4.3. Leukocyte-Derived Vesicles

Cells of the innate and the adaptive immune system, including T and B cells, dendritic cells, monocytes and macrophages, mast cells, natural killer cells, and polymorphonucleate cells release both MVs and exosomes.

Leukocyte-derived EVs usually contain inflammatory cytokines (e.g., interleukine 1 beta), intracellular cell adhesion molecule-1 (ICAM-1), P-selectin glycoprotein ligand-1 (PSGL-1), TF, complement receptor 3 (C3), metalloproteases [97,98,99,100], and nucleic acids (tRNAs, mRNAs, miRNAs, and long noncoding RNAs) [101]. However, EVs from different subpopulations of leukocytes differ in the composition of the plasma membrane as well as in cytosolic proteins. For instance, monocyte-derived EVs specifically express CD11b, CD14, CD64 and CD142; CD3 and CD45 are associated with lymphocyte-derived EVs, whereas EVs released from neutrophils present CD35, CD66b and myeloperoxidase [58,102] (Figure 2).

Leukocyte-derived EVs promote leukocyte activation and trans-endothelial migration [100,103], modulate specific immune responses, inflammatory reactions, atherogenesis, plaque rupture and thrombosis [87,104]. For instance, during the innate immune response to bacterial infections, activated platelets bind to neutrophils and stimulate the release of EVs that contain arachidonic acid that in turn is delivered to platelets to support the generation of proinflammatory and proaggregating mediators [105].

Exosomes derived from monocytes stimulated with IFNα or LPS are specifically enriched in miR-222, miR-155, miR-146a, miR-146b, and miR-125a-5p. By contrast, miR-222 is significantly down-regulated in exosomes derived from monocytes treated with both IFNα and LPS [106]. Similarly, the composition of exosomes released by neutrophils changes upon LPS stimulation [107], and, as macrophages-derived exosomes, display a high expression level of miR-21-5p, and miR-155-5p [108,109] (Table 2).

### 4.4. Endothelial-Derived Vesicles

Endothelial cells release MVs (EC-MVs) during cellular activation and ApoBDs during apoptotic cell disassembly. EC-derived EVs are physiologically relevant since they regulate endothelial cell survival [110], but they are also involved in several pathological processes [21,111]. In healthy individuals, EC-MVs account for approximately 5–15% of EVs in peripheral blood [111].

Circulating EC-MVs are characterized by endothelial cell markers: CD31, CD62E, CD144, CD105, and VCAM-1 [112,113] (Figure 2). In addition, EC-MVs show increased surface expression of PS and TF, which partially explains their procoagulant activity [114], and other proteins, as reported in Table 2 [114,115]. ApoBDs generated from endothelial cells during apoptosis share the same markers of EC-MVs but are easily distinguishable from the latter due to their larger size.

Protein and RNA profiles of exosomes secreted by ECs are affected by cellular stresses [116,117]. Moreover, depending on the cellular context, endothelial cells secrete exosomes enriched in specific miRNAs, such as miR-214, miR-210, miR-126, and miR-146a [118,119,120] (Table 2).

## 5. Involvement of EVs in Arterial and Venous Thrombosis

### 5.1. Effect of EVs on Thrombosis

The contribution of EVs, in particular of MVs, to thrombotic events is due to their procoagulant surface and to the expression of highly procoagulant proteins, such as TF. MVs formation leads to the externalization of anionic phospholipids, mainly PS, that significantly contribute to procoagulant activity associated with MVs. Indeed, the externalized negative phospholipids favor the assembly and the activation of tenase and prothrombinase complexes, thus potentiating thrombin formation [121]. Although PS is exposed on the surface of most MVs, particularly those of platelet origin [70,71], a group of MVs negative for annexin V staining has also been detected, demonstrating the existence of PS-negative MVs population [122].

The procoagulant activity of some classes of MVs is further increased by the expression of TF, and MVs harboring both PS and TF have the highest procoagulant activity. TF is a key activator of the coagulation cascade; its extracellular domain binds and activates FVII, triggering hemostasis after vascular injury, and its aberrant activation causes thrombosis [123]. Under physiological conditions TF is expressed by cells surrounding blood vessels, such as fibroblasts of the tunica adventitia, to constitute a hemostatic envelope; conversely, it is not constitutively expressed by cells in contact with blood, such as endothelial cells, neutrophils, lymphocytes, and monocytes which, however, can transiently express it upon activation [124]. In addition, it has been shown that also human circulating platelets and their precursor cell megakaryocytes express TF [72,74,125]. However, some authors fail to detect TF in these types of cells [126,127,128,129]. Methodological differences in sample preparation may explain this discrepancy [126,130].

A low amount of TF is present in the blood of healthy individuals. This form of circulating TF, called “blood-borne TF” [128,131], is mainly associated with MVs originating from both vascular and circulating cells [115,132], including monocytes and platelets [72,115,131,133]. The exposure of vessel wall-derived TF at the site of vascular injury would play its main role in the initiation phase, whereas the blood-borne TF would be involved in the propagation phase of thrombus formation [134,135]. Of note, several studies documented that the physiological levels of blood-borne TF can increase in pathological conditions characterized by a prothrombotic phenotype.

Most of cell surface-exposed TF is in an encrypted state and it requires activation to fully exhibit its procoagulant potential. Although these molecular mechanisms are not completely understood, the exposure of PS induced by different stimuli, dissociation of TF dimers into monomers, and disulfide bond switching represent critical events in TF decryption [136]. Activated platelets may induce activation of TF by secreting protein disulfide isomerase (PDI), which mediates TF decryption through the redox switch of an exposed disulfide bond in TF extracellular domain [137,138]. In addition, since TF^+^MVs are able to bind activated platelets [139], it is reasonable that locally released PDI activates TF exposed on MVs membrane and further amplifies the hemostatic process.

Of note, in a particular setting, the amount of active TF on MVs membrane is higher than that of parental cell. Thomas et al. demonstrated in vivo that cancer cell-derived MVs, but not their parent cells, reduced tail bleeding time and the time to occlusion of venules and arterioles [140]. Similarly, Geddings and colleagues [141] showed that cancer cell-derived TF^+^ MVs enhanced blood coagulation and induced platelets aggregation in mice.

Circulating MVs can contribute to thrombosis also through indirect mechanisms, independents of TF and PS surface expression that promote intercellular communications. Ex vivo studies performed in the Badimon chamber have shown that the enrichment of human blood with MVs isolated from healthy subjects significantly increased platelet deposition on damaged arteries [142]. Similarly, blood enriched with PMPs induced fibrin deposition on human atherosclerotic arteries and platelet adhesion to collagen-coated surfaces. PMPs shortened epinephrine/collagen closure time evaluated by PFA-100, increased platelet aggregation in response to low doses of ADP and reduced clotting time. These observations suggest that PMPs, even under normal blood conditions, enhance platelet activation and thrombus formation [142]. In addition, MVs released from activated platelets may, in turn, induce activation of other platelets by transferring arachidonic acid [143].

Circulating MVs can also indirectly activate the coagulation cascade. Van Der Meijden and colleagues [144] showed that coagulation of Factor VII-deficient plasma cannot be initiated by monocyte-derived MVs, establishing that these MVs trigger coagulation predominantly via TF. Moreover, erythrocyte- and platelet-derived MVs failed to activate coagulation in Factor XII-deficient plasma, suggesting that these MVs can induce thrombin generation in a Factor XII-dependent manner [144]. Similarly, the ability of MVs released from LPS-stimulated monocytic cell line to induce thrombin generation is efficiently blocked by anti-TF antibodies [145]. Coagulation may also be promoted by erythrocyte-derived MVs in Factor XI-dependent manner, as shown in hemolytic disorders such as sickle cell anemia [146,147].

Moreover, the exposure of endothelium to both microvesicles and exosomes isolated from monocytes resulted in the overexpression of TF on endothelial cells surface and in the reduction of anticoagulant tissue factor pathway inhibitor (TFPI) and thrombomodulin (TM), suggesting that monocyte-derived MVs increase endothelial thrombogenicity [148]. On the other hand, EC-MVs stimulated TF expression and increased the procoagulant activity in monocytic cell line THP-1 [149].

In addition to their procoagulant properties, EVs may affect coagulation through anticoagulant or fibrinolytic mechanisms. In vitro studies showed that erythrocyte-derived and platelet-derived MVs bound protein S and supported the anticoagulant activity of activated protein C [150,151]. Moreover, EC-MVs stimulated fibrinolysis through an autocrine mechanism. In particular, the urokinase-type plasminogen activator receptor (uPAR), expressed on the surface of EC-MVs, enhanced the activation of plasminogen [152]. The same authors showed that only microvesicles generated from endothelial cells and leukocytes, but not those from platelets or erythrocytes, sustained plasmin generation, suggesting that only these classes of MVs may support fibrinolytic activity in the circulation [153]. Moreover, MVs harbor functionally active TFPI that counterbalances TF activity, preventing its abnormal activation [154].

Finally, exosomes released from activated platelets reduced CD36 in platelets and macrophages through ubiquitination and proteasome degradation, with the consequent decrease in platelet aggregation and adhesion, thus preventing thrombus formation [155]. In addition, platelet-derived exosomes carrying miR-320 promoted endothelial cell mobility, decreased inflammation and thrombus formation reducing expression of ICAM-1 in endothelium [156]. Interestingly, exosome released by mast cells stimulated expression and activity of plasminogen activator inhibitor type 1 in endothelial cells contributing to thrombosis and atherosclerosis [157].

In conclusion, depending on their parental cell, EVs actively participate in the regulation of the delicate balance between coagulation and fibrinolysis.

### 5.2. Pathological Function of EVs in Animal Models of Arterial and Venous Thrombosis

The contribution of MVs to arterial thrombosis has been largely investigated in mice models, mainly focusing on the interaction between MVs and platelets to promote thrombosis. However, knowledge obtained from the murine models needs to be carefully translated to human taking account of the differences between these two species [158,159].

In a laser-induced arterial injury model, Falati et al. [139] have shown that TF^+^ MVs derived from monocytes were recruited in the growing thrombi, where they participate to fibrin generation. The accumulation of TF^+^ MVs in the thrombi was promoted by the interaction of PSGL-1 on MVs with platelet P-selectin. The critical role of PSGL-1 and P-selectin in this scenario was confirmed by the limited accumulation of TF in the thrombus of PSGL-1 or P-selectin null mice [139]. Similarly, in wild type mice, TF recruitment was prevented by injection of P-selectin blocking antibody [139].

In line with the key role of MV–platelet interaction in arterial thrombosis, Ghosh et al. [160] have demonstrated, in a FeCl_3_-induced injury model, that thrombus formation was supported by the accumulation of EC-MVs through their binding to platelet CD36. Indeed, in CD36 null mice, a longer time to occlusion was observed and the accumulation of EC-MVs in arterial thrombi was diminished compared to wild type control mice [160].

In addition, EVs released from macrophages upon ATP stimulation modulated procoagulant activity and arterial thrombosis in mice [161].

Studies investigating the contribution of MVs to venous thrombosis are limited and mainly performed in an inferior vena cava (IVC) ligation stasis mouse model. However, the use of ligation stasis has the disadvantage to partially limit the delivery of MVs to the site of thrombus, and thus the thrombus features in the clinical scenario might not be fully reproduced by this model [162].

In mice undergoing IVC ligation, MVs isolated from the animal with thrombus have higher TF-associated activity compared to animals without thrombus [163,164]. The injection of MVs isolated from thrombosed animals into separate mice undergoing IVC ligation positively impacts on thrombus weight [163], suggesting that MVs play a key role to sustain venous thrombogenesis. Interestingly, TF^+^MVs activity positively correlated with both thrombus size and urinary 2,3-dinor-TXB_2_, and aspirin treatment concomitantly decreased the activity of TF associated with MVs as well as thrombus size [164], providing a link among platelets, TF^+^MV activity, and venous thrombosis.

Likewise, Birò et al. [165] have shown that MVs isolated from pericardial blood of cardiac surgery patients were highly thrombogenic in a venous stasis thrombosis rat model, compared to microvesicles isolated from healthy subjects, and that MVs thrombogenicity was abolished by an antibody against TF. In these patients, the total number of TF^+^MVs was higher than in healthy subjects. In particular, the percentage of PMPs exposing TF was significantly higher, whereas the concentration of TF^+^MVs derived from erythrocytes and granulocytes did not differ between the two groups. These results sustain the key thrombogenic role of PMPs exposing TF and suggest their involvement in the pathological conditions characterized by increased thromboembolic tendency.

#### Pathological Function of EVs in Cancer-Associated Thrombosis

Even though the pathogenesis of cancer-associated VTE is quite different from general VTE, several studies have explored the role of MVs in cancer-associated VTE showing that tumor cell-derived MVs promoted coagulation and thrombus formation in vivo in TF-dependent manner [166]. In vivo studies showed that cancer cells released procoagulant MVs into the circulation, which was TF- and PS-dependent. In particular, the procoagulant activity associated with MVs-released from breast cancer, pancreatic cancer, and melanoma cells was completely abolished by anti-TF antibodies or annexin V [167,168].

Moreover, pancreatic and lung cancer cell-derived MVs carrying TF and PSGL-1 promoted platelet aggregation sustaining thrombus growth [139,140]. In vivo, cancer cell-derived MVs infused in mice accumulated at the site of injury in a P-selectin-dependent manner. These MVs reduced tail bleeding time and the time of occlusion of venules and arterioles, likely through the MVs-associated TF. Mice bearing a tumor under-expressing TF, thus presenting low circulating levels of TF activity, had an increased bleeding time and a 100-fold diminished fibrin generation and platelet accumulation at the site of injury [169].

In this context, Wang et al. [170] showed that only mice with TF-positive tumors had elevated levels of TF^+^MVs and showed enhanced thrombosis in a saphenous vein FeCl_3_ injury model. By contrast, the contribution of cancer-derived TF^+^ MVs to thrombus formation in the IVC stenosis mouse model is under debate. Wang et al. [170] demonstrated that TF^+^ MVs released from tumors were insufficient to trigger venous thrombosis in tumor-bearing mice subjected to IVC stenosis, whereas Thomas et al. [171] provided evidence that all tumor-bearing mice formed an occlusive thrombus after 3 h of stenosis. The reason for these conflicting results is likely due to differences in both the IVC stenosis model used and in the circulating levels of TF^+^ MVs produced by tumor cells. In fact, Wang et al. [170] using exogenous cancer-derived TF^+^ MVs suggested that the level of TF^+^ MVs required to increase venous thrombosis was 40 times greater than those detected in their tumor-bearing mice. In addition, Thomas et al. [171] carried the experiments in C57BL/6 mice bearing mouse pancreatic tumors (Panc02) whereas Wang et al. [170] used nude mice (BALBc background) bearing human pancreatic tumors (HPAF-II), thus suggesting that the different mouse genetic backgrounds, as well as cancer cells used in the two studies may explain the discrepancy.

Finally, it was proposed that exosomes derived from tumor cells accelerate venous thrombosis in vivo by inducing the release of neutrophil extracellular traps (NETs) from circulating neutrophils and by interacting with them [171,172].

Studies investigating the role of EVs in thrombosis are summarized in Table 3.

## 6. Clinical Applications

Besides their relevant roles in intercellular communication and their contribution in the thrombotic manifestation of several pathological conditions, including thrombosis and cardiovascular diseases, EVs represent an attractive diagnostic tool for a noninvasive liquid biopsy. Indeed, during their biogenesis, EVs incorporate proteins, lipids, and coding and noncoding RNAs from their parental cells, potentially acting as a pathophysiological signature of cellular and tissue activation/modification.

The analyses of EVs, in terms of counts, surface marker expression, protein and miRNA cargo, have generated promising results for diagnosis, prognosis, and therapeutic monitoring in several clinical settings, including atherosclerosis, acute coronary syndrome, deep vein thrombosis and pulmonary embolism [9,102,173,174,175,176,177,178,179,180,181,182,183,184,185,186,187,188].

In addition, given the involvement of EVs in disease pathogenesis, novel therapeutic options should consider targeting EVs. Blockage of EVs release and/or their interaction with target cells can be achieved in various ways, mainly by inhibiting the vesicle release, uptake, or formation [189].

### 6.1. EVs as Biomarkers in Arterial Thrombosis

Higher levels of EVs from leukocytes, including lymphocytes and monocytes, have been detected in patients with acute coronary syndrome (ACS) in the first hours after the event [190,191], and they were associated with cardiovascular disease severity and mortality [73,192].

Similarly, EVs from erythrocytes increase in whole blood of STEMI patients after primary angioplasty. These MVs have a different pattern of distribution compared to healthy individuals and are positively associated with adverse clinical events [80].

Interestingly, EC-derived EVs also displayed a good prognostic value for the occurrence of cardiovascular events, reflecting the status of the damaged endothelium. Moreover, in coronary artery disease (CAD) patients, CD31^+^/Annexin V^+^ EC-EVs have been associated with a worse clinical outcome, including an increased incidence of adverse cardiovascular and cerebral events [193]. Likewise, in acute myocardial infarction (AMI) the EC-EVs positively correlated with the myocardium at risk and with infarct size, as well as with troponin levels, and were inversely associated with left ventricular ejection fraction value [194]. Elevated plasma levels of EC-EVs have been associated with unstable asymptomatic carotid plaques [195]. In patients with heart failure, plasma ratio of CD31^+^/Annexin V^+^ EC-EVs and mononuclear progenitor cells, as well as the high levels of CD144^+^-EC-EVs are an independent predictor for adverse cardiovascular events [196,197].

The studies carried out over time to evaluate the association between PMPs and cardiovascular diseases produced different results. Indeed, some studies have shown that the plasma levels of PMPs were higher in patients with cardiovascular diseases compared to healthy subjects [176,183,188,198].

In particular, high levels of PMPs bearing P-selectin have been strongly associated with future atherothrombotic events within two years [73,199]. By contrast, others reported no difference in circulating levels of these PMPs, although they observed an increased in both erythrocyte-MVs and TF^+^MVs in myocardial infarction patients treated with primary angioplasty and with ST-segment elevation, respectively [173,200]. However, a positive correlation between plasma levels of PMPs and increased risk of ACS was recently found in a systematic review and meta-analyses that analyzed 449 patients with ACS, 93 with stable angina, and 192 healthy controls. The authors showed that percutaneous coronary intervention can reduce circulating levels of PMPs [201], concluding that these MVs might be good predictor and prognostic factors of ACS.

In addition, in patients with familial hypercholesterolemia, the levels of PMPs correlated with lipid-rich atherosclerotic plaques and inversely with calcified plaques, suggesting their usefulness as potential biomarkers for the prediction of plaque vulnerability [190].

Interestingly, ex vivo and in vivo experiments showed that the release of MVs from platelets was strongly influenced by antiplatelet drug treatments.

In particular, cangrelor, prasugrel, or clopidogrel prevented PMPs release induced by thrombin receptor activating peptide (TRAP) or collagen and/or inhibited their procoagulant activity [202,203]. Similar results have been obtained after intravenous administration of cangrelor [203]. These observations are consistent with the clinical studies revealing a negative correlation between serum levels of clopidogrel and PMPs in stable CAD patients [204], and showing a reduction in PMPs in ACS patients following the treatment with clopidogrel [188]. Preliminary data from the TIGER-M clinical study showed that ticagrelor had a similar effect to clopidogrel in reducing PMP levels in non-STEMI patients [205,206]. Interestingly, in ACS patients the levels of PMPs reflected the clinical response to clopidogrel. Indeed, patients with high-on treatment platelet reactivity had higher levels of PMPs compared to clopidogrel responder patients who have significantly lower PMPs [207], suggesting that PMPs might be helpful tools to monitor antiplatelet therapy in CAD patients.

In contrast to the reported effect of P2Y12 inhibitors, the impact of aspirin on the release of PMPs produced different results. Bulut et al. [208] showed that aspirin treatment for eight weeks lowered PMP levels in stable CAD patients. Ex vivo study showed that preincubation with aspirin reduced PMPs induced by arachidonic acid, collagen and TRAP-6 but did not affect the response to epinephrine or ADP [209]. On the other hand, the same aspirin dosage did not affect PMP concentrations in patients with type 2 diabetes, or arterial fibrillation, acute ischemic stroke, as well as healthy subjects [210,211,212,213]. Overall, these data suggest that aspirin may differently affect PMPs behavior according to the pathology. Finally, Abiciximab treatment significantly reduced PMP levels in STEMI patients with primary percutaneous coronary intervention compared to patients who did not received this drug, whereas no effect was observed in patients treated with eptifibatide [214].

Besides the alteration in the concentration of the different classes of EVs, the analysis of their content in terms of protein and miRNA may provide further insights into the pathophysiological mechanisms occurring in cardiovascular diseases. In particular, EVs protein and miRNA expression profile have been associated with cardiovascular disease.

Proteome analysis showed that EVs isolated from plasma of STEMI patients express higher levels of several proteins involved in thrombogenesis (e.g., α2-macroglobulin isoforms, fibrinogen, and viperin) compared to stable CAD patients [215]. The protein profile of EVs isolated from patients with MI showed marked differences in terms of complement activation pathways (Complement C1qA and Complement C5), lipoprotein metabolism (Apolipoprotein D and Apolipoprotein C-III) and platelet activation (platelet glycoprotein Ib alpha chain and platelet basic protein) when compared to those of patients with stable angina [216]. Similarly, high levels in EVs of proteins involved in inflammatory processes, such as SerpinF2 and SerpinG1, have been related to the occurrence of heart failure. In addition, exosomes isolated from ACS patients had a different proteomic profile compared to non-ACS patients. Interestingly, higher levels of polygenic immunoglobin receptor, cysteine C and complement factor C5 in EVs have been associated with ACS [217].

In addition, several studies report that patients with AMI and unstable angina have altered levels of TF^+^ MVs [218,219]. However, other works failed to detect an increase in the concentration of TF^+^MVs [220,221]. These incongruences might be due to the incorporation/adherence of MVs in/to the coronary thrombus that may lead to a reduction in circulating MVs.

Finally, in patients with familial hypercholesterolemia, TF^+^ EVs positively correlated with lipid-rich atherosclerotic plaques and inversely with calcified plaques [190]. High levels of monocyte-derived TF^+^MVs, potentially induced by oxidized LDL [198], were also detected in atherosclerotic plaques [182]. The higher thrombogenic capacity of intra-atherosclerotic plaques TF^+^MVs compared to circulating MVs suggests their contribution in the induction of thrombosis upon plaque rupture [182].

Likewise, a greater amount of several miRNAs, including miR-208a, miR-133a, and miR-499, has been detected in exosomes isolated from ACS patients, and a negative association between exosomes-miR-208a levels and survival rate was found [222,223]. Of note, increased expression of miR-126 and miR199a mainly associated with both PMPs and EC-MVs, but not their freely circulating form, predict the occurrence of cardiovascular events in patients with stable CAD [224]. In addition, higher expression of proteins involved in inflammatory processes, such as miR-199b-3p, miR-27b-3p, miR-130a-3p, miR-221-3p, and miR-24-3p in exosomes of patients with asymptomatic carotid artery stenosis are associated with stenosis progression [225].

Interestingly, Emanueli et al. showed that troponin I levels positively correlated with miR-1, miR-24, miR-133a, and miR-133b contained in exosomes isolated from plasma of coronary-artery-by-pass-graft (CABG) patients at 24 and 48 h postsurgery [226]. Finally, in post-AMI patients who developed heart failure within one year of AMI onset, serum levels of three exosome-associated miR-192, -194, and -34a were good predictors of heart failure development [184]. Therefore, circulating EVs can be a potential source of biomarkers for early prevention and diagnosis of cardiovascular events.

### 6.2. EVs as Biomarkers in Venous Thrombosis

The diagnostic value of EVs in venous thrombosis has been poorly studied. Recurrent DVT is associated with increased levels of endothelial- and monocyte-derived MVs, compared to patients suffering from an initial DVT event [227]. However, most of the studies on EVs and venous thrombosis have been performed in patients with cancer or in mouse cancer models, in virtue of the higher risk of these patients of developing VTE [228,229,230,231]. Only little information is available concerning the relationship between EVs and non-cancer-related venous thrombosis.

Many studies focused the attention on the role of TF^+^ MVs, and almost all concluded that TF^+^ MVs and their activity are increased in VTE patients [178,227,232,233,234], with the except of Steppich and collaborators [235], who did not find any association between circulating TF and MVs in patients with deep vein thrombosis.

VTE risk is increased in other syndromes, such as autoimmune disorders (e.g., Behçet’s Syndrome, inflammatory bowel disease), pathologies with deficiencies of natural anticoagulants, and systemic lupus erythematosus. As expected, an increase in the concentration of different classes of MVs, in particular of TF^+^ MVs, has been detected in these classes of patients [236,237,238,239].

In addition, a hyperthrombotic state associated with an increase of TF^+^ MVs has also been detected in some infectious diseases, such as bacterial sepsis. In this pathological context, bacterial endotoxin stimulated monocytes to release high levels of TF^+^ MVs, that are the main inducer of the prothrombotic manifestation in those patients [148,240]. In patients with heparin-induced thrombocytopenia, a positive correlation between higher levels of leukocyte-derived TF^+^ MPs and hyper thrombotic risk has been found [241]. In hemolytic disorders (e.g., sickle cell anemia, thalassemia) the hypercoagulable state has been associated with Factor XI-dependent procoagulant properties of erythrocyte-MVs [146,147].

Moreover, the levels of circulating PMPs were significantly higher in patients with pulmonary thromboembolism (PTE) compared to both healthy controls and patients with suspicious PTE, suggesting that PMP level has a predictor value for PTE, and a diagnostic accuracy similar to D-dimer. Remarkably, the combination of PMPs, platelet distribution width, P-selectin and D-dimer exhibited high sensitivity, specificity, and accuracy in the diagnosis of PTE [242]. The key role of PMPs in venous thrombosis has been demonstrated in IVC ligation animal model [163]. Indeed, in this experimental model, thrombus weight correlated negatively with MVs derived from leukocytes, and positively with MVs derived from platelets [163].

Finally, Jamaly et al. showed that patients with unprovoked VTE had higher plasma concentrations of PSGL-1-bearing MVs than control subjects; however, large population-based prospective studies are required to validate these findings [243].

#### EVs as Biomarkers in Cancer-Associated Thrombosis

In this context, particular attention needs to be paid to the relationship between procoagulant MVs, VTE, and cancer.

Several solid and hematologic tumors express TF, whose level of expression correlates with thromboembolic complications and poor prognosis [231]. As for the other cell types, cancer cells may release TF-bearing MVs [244,245].

Independent clinical studies found a greater amount of TF^+^ MVs and higher TF activity associated with MVs in cancer patients with venous thrombosis compared to patients without venous thrombosis [246,247,248,249,250] and showed that cancer patients with higher TF^+^ MVs levels have a higher risk of developing VTE [251,252]. Nevertheless, the incidence values for VTE differs according to the types of cancer.

It has been suggested that the procoagulant activity of MVs can be used to predict venous thromboembolism in cancer patients [253]. A recent meta-analysis including six studies (four cohort studies and two case-control studies) summarized that TF-bearing MVs were associated with increased risk of VTE in cancer patients [108]. However, the lack of association between circulating TF^+^ MVs or MVs-TF activity and VTE in small cell lung, stomach, or colorectal cancer, and in multiple myeloma patients were found [254,255,256], limits the feasibility of TF^-^MVs as predictive factors of VTE risk in cancer patients. Cancer heterogeneity may explain these differences. Indeed, a strong relationship between MP-TF activity and VTE in pancreatic cancer patients was observed but not in other types of cancers [257].

### 6.3. Potential Therapeutic Application of EVs in Cardiovascular Diseases

The potential application of EVs in the clinical setting also includes their use as therapeutic delivery tools. EVs stimulate tissue regeneration and have cardioprotective properties [258,259]. Preclinical studies demonstrated that treatment with conditioned media of hypoxic mesenchymal stem cells (MSCs) decreased infarct size and improved cardiac function after MI in mouse and pig models [260,261] and a similar effect was observed using MSC exosomes [262]. In addition, EC-MVs generated in vitro reduced atherosclerotic lesion by transferring miR-143/145 to smooth muscle cells [263].

Furthermore, a promising therapeutic application of EVs is their use as a drug delivery system to increase solubility, stability, and bioavailability of drugs in the blood circulation. Sun et al. [264] demonstrated that exosomes derived from endothelial progenitor cells (EPCs) loaded with miR-126 promoted migration and angiogenesis of EPCs and contributed to thrombus resolution in a mouse model of venous thrombosis.

Therapeutic innovation in the field of EVs includes the construction of synthetic MVs mimicking the natural ones. For instance, Pawlowski et al. [265] designed nanovesicles mimicking PMPs able to accumulate at the site of clot formation and to deliver thrombolysis.

## 7. Conclusions

The putative role of EVs in hemostasis and thrombosis is supported by a large number of studies unraveling how these vesicles affect the thrombotic processes. Prothrombotic characteristics of MVs mainly rely on the expression of both PS and TF on their surfaces. Moreover, the presence on MVs surface of other molecules and receptors, including PSGL-1 and GPIIb/IIIa, further enhances coagulation and thrombosis.

Noteworthy, thrombotic disorders are often associated with altered levels of the different classes of EVs, thus suggesting their potential use as biomarkers. The presence of EVs in all body fluids, such as blood and urine, makes them an attractive tool for noninvasive liquid biopsy. The number of circulating MVs, as well as their protein and miRNA content, may reflect disease prognosis and severity and may predict future events. Moreover, EVs have been demonstrated to play a role in tissue regeneration and fibrinolysis, and given their ability to mediate cell to cell communication, EVs may be exploited as a drug delivery system.

Despite the promising results, the emerging potential of EVs as biomarkers, a delivery drug system, or mediators of regenerative mechanisms still needs to overcome some limitations. First, most clinical studies included a relatively low number of subjects; therefore, such observations must be validated in larger cohorts of patients. Another relevant issue is the limited knowledge about the effect of concurrent use of multiple medications on circulating EVs. Indeed, it is well known that some antiplatelet drugs, antihypertensive agents, and statin therapy may influence shedding and composition of MVs [6]. Finally, the several purification methods to isolate EVs subtypes (e.g., differential ultracentrifugation, density gradient centrifugation, size exclusion chromatography) lead to EV preparation of different composition and purity. Therefore, to obtain reliable conclusions on the role of EVs in physiological and pathological conditions or on their use as biomarkers, it is crucial to follow the recently published official guidelines [7,8,9,10,11].

In conclusion, advancing our knowledge about mechanisms of EVs formation and their pathophysiological relevance may help to shed light on circulating EVs and to translate their application to clinical practice.

## Figures and Tables

**Table 1:** Methods for the isolation and the characterization of MVs and exosomes.

Type of EVs	Biogenesis	Isolation	Detection
**Microvesicles (100 nm–1 µm)**	Blebbing of plasma membrane	Ultracentrifugation, density gradients	AFM, EM, NTA, IF, FC, ELISA
**Exosomes (30–100 nm)**	Release by exocytosis of MVBs	Ultracentrifugation, immunopurification, density gradient, commercial kit, size exclusion chromatography	AFM, EM, NTA, RPS, DLS, WB, ELISA
**Apoptotic body (500 nm–4 µm)**	Blebbing of apoptotic cells	Centrifugation, filtration, FACS	IF, FC

MVBs: multivesicular bodies; AFM: atomic force microscope; EM: electron microscopy; NTA: nanoparticle tracking analysis; IF: immunofluorescence microscopy; FC: flow cytometry; ELISA: enzyme-linked immunosorbent assay; RPS: resistive pulse sensing; DLS: dynamic light scattering; WB: western blotting; FACS: fluorescence activated cell-sorting.

**Table 2:** Proteins and miRNA usually associated with MVs and exosomes.

	MVs	Exosomes
**Platelets**	GPIb, TF, CD31, CD36, CD62P, CD61, CD40L, vWF, fibrinogen, thrombospondin	miR126-3p, mi-R21, mi-223, miR-339, miR-328, miR-22, miR-185, miR-320b, GPIb, GPV, CXCL4, CXCL7, HMGB1
**Megakaryocytes**	GPVI, CD42b	
**Erythrocytes**	Band 3, Actin, Hemoglobin A, CD55, CD59, Iron, Annexin A1, annexin A2, glut1	
**Leukocytes**	ICAM-1, TF, PSGL-1, CD62L, C3, MMPs, inflammatory cytokines	miR-222, miR-155, miR-146a, miR-146b and miR-125a-5p, miR-21-5p
**Endothelial cells**	Annexin A1, annexin A2, actin, cofilin, calnexin, calreticulin, caveolin-1, thrombospondin, CD59, ICAM-1, α5β1, α2β1	miR-214, miR-210, miR-126, miR-146a, MiR-206, ARF6, NCX1

GP: glycoprotein; TF: tissue factor; vWF: Von Willebrand factor; CXCL: chemokine (C-X-C motif) ligand; HMGB1: High mobility group box 1; ICAM-1: Intracellular adhesion molecule 1; CD62L: l-selectin; MMP: metalloproteinase; PSGL-1: P-selectin glycoprotein ligand-1.

**Table 3:** Summary of studies investigating the role of EVs in thrombosis.

Type of EVs	Major Findings	Reference
**Platelet-derived EVSs**	PMPs induce fibrin deposition on atherosclerotic arteries. Increase platelet aggregation and adhesion to collagen. Shorten epinephrine/collagen closure and reduce clotting time	[142]
	PMPs induce trans-activation of platelets by transferring arachidonic acid	[143]
	PMPs promote thrombin generation in a Factor XII-dependent fashion	[144]
	PMPs bind protein S and support the anticoagulant activity of activated protein C	[151]
	Platelet-exosomes inhibis platelet activation, endothelial mobility, inflammation and proatherothombotic cellular functions	[155,156]
**Erythrocytes-derived MVs**	Induce coagulation through a factor XI- and factor XII-dependent mechanism	[144,146,147]
	Bind protein S and support the anticoagulant activity of activated protein C	[150,151]
**Leukocyte-MVs**	Sustain plasmin generation	[153]
**Mast cell-exosome**	Stimulate expression and activity of plasminogen activator inhibitor type 1	[157]
**Monocyte-MVs**	Trigger coagulation predominantly via TF	[144,145]
	Induce overexpression of TF and the reduction of TFPI and TM on endothelial cells	[148]
	Participate to fibrin generation and thrombus growth in vivo	[139]
**Macrophages-EVs**	Modulate procoagulant activity and arterial *thrombosis* in vivo	[161]
**EC-MVs**	Stimulate TF expression and procoagulant activity in monocytic cell line	[149]
	Enhance plasminogen activation, plasmin generation and fibrinolysis	[173]
	Bind to platelet CD36 and support thrombus formation in vivo	[160]
**Cancer cell-EV**	Reduce bleeding time and time of vessel occlusion	[140]
	Cancer cell-MVs enhanced blood coagulation and platelet aggregation	[141]
	Promote TF-dependent coagulation and thrombus formation in vivo	[166,167,168,169,170]
	Cancer cell-Exosomes accelerate venous thrombosis in vivo by inducing the release of NETs	[171,172]

## Abbreviations

ACS	Acute coronary syndrome
AFM	Atomic force microscope
AMI	Acute myocardial infarction
ApoBD	Apoptotic body
ARF6	Adenosine diphosphate-ribosylation factor 6
ALIX	ALG-2 interacting protein X
ARRCD1	Arrestin domain-containing protein-1
ATP	Adenosine triphosphate
C3	Complement receptor 3
CABG	Coronary-artery-by-pass-graft
CAD	Coronary artery disease
CD62L	L-selectin
CXCL	Chemokine (C-X-C motif) ligand
DLS	Dynamic light scattering
DVT	Deep vein thrombosis
EC-MV	Endothelial cell microvesicle
ELISA	Enzyme-linked immunosorbent assay
EM	Electron microscopy
EPC	Endothelial progenitor cell
ERK	Extracellular signaling-regulated kinase
ESC	European Society of Cardiology
ESCRT	Endosomal sorting complex required for transport
EV	Extracellular Vesicle
EXO	Exosome
FACS	Fluorescence activated cell sorting
FC	Flow cytometry
FeCl_3_	Ferric chloride
GPIb	Glycoprotein Ib
GPIIb/IIIa	Glycoprotein IIb/IIIa
GPV	Glycoprotein V
GPVI	Glycoprotein VI
GTP	Guanosine-5′-triphosphate
HMGB1	High mobility group box 1
HSP70	Heat shock proteins 70 Kd
ICAM-1	Intracellular adhesion molecule 1
IF	Immunofluorescence microscopy
ILV	Intraluminal vesicle
INFα	Interferon α
ISEV	International Society for Extracellular Vesicles
ISTH	International Society on Thrombosis and Haemostasis
IVC	Inferior vena cava
LDL	Low-density lipoprotein
LPS	Lipopolysaccharide
MI	Myocardial infarction
MLC	Myosin light-chain
MLCK	Myosin light-chain kinase
MMP	Metalloproteinase
MSC	Mesenchymal stem cell
MV	Microvesicle
MVB	Multivesicular body
NET	Neutrophil extracellular trap
NTA	Nanoparticle tracking analysis
PDI	Protein disulfide-isomerase
PE	Pulmonary embolism
PFA-100	Platelet function analyzer
PKC	Protein kinase C
PLD	Phospholipase D
PMP	Platelet-derived microparticle
PS	Phosphatidylserine
PSGL-1	P-selectin glycoprotein ligand-1
PTE	Pulmonary thromboembolism
RNA	Ribonucleic acid
RPS	Resistive pulse sensing
STEMI	ST elevation myocardial infarction
TEM	Transmission electron microscopy
TF	Tissue factor
TFPI	Tissue factor pathway inhibitor
TM	Thrombomodulin
TNF-α	Tumor necrosis factor α
TRAP	Thrombin receptor activating peptide
TSG101	Tumor susceptibility gene 101
TXB_2_	Thromboxane B_2_
uPAR	Urokinase-type plasminogen activator receptor
VCAM-1	Vascular cell adhesion protein 1
Vps-4	Vacuolar protein sorting-associated protein 4
VTE	Venous thromboembolism
WB	Western blotting
